# A strongly selected mutation in the HIV-1 genome is independent of T cell responses and neutralizing antibodies

**DOI:** 10.1186/s12977-017-0371-4

**Published:** 2017-10-10

**Authors:** Donglai Liu, Chu Wang, Bhavna Hora, Tao Zuo, Nilu Goonetilleke, Michael K. P. Liu, Mark Berrong, Guido Ferrari, Andrew J. McMichael, Tanmoy Bhattacharya, Alan S. Perelson, Feng Gao

**Affiliations:** 1 National Engineering Laboratory for AIDS Vaccine, School of Life Sciences, Jilin University, Changchun, Jilin China; 2 Department of Medicine, Duke Human Vaccine Institute, Duke University Medical Center, 303 Research Dr., 244 Sands Building, DUMC 102359, Durham, NC 27710 USA; 3 Department of Microbiology, Immunology and Medicine, University of North Carolina at Chapel Hill, Chapel Hill, NC USA; 4 Weatherall Institute of Molecular Medicine, University of Oxford, Oxford, England, UK; 5 Department of Surgery, Duke University Medical Center, Durham, NC USA; 6 Theoretical Division, Los Alamos National Laboratory, Los Alamos, NM USA; 7 Present Address: Division II of In Vitro Diagnostics for Infectious Diseases, Institute for In Vitro Diagnostics Control, National Institutes for Food and Drug Control, Beijing, China

**Keywords:** Mutation, Selection, Immune responses, Cryptic T cell response, Fitness, Escape

## Abstract

**Background:**

Mutations rapidly accumulate in the HIV-1 genome after infection. Some of those mutations are selected by host immune responses and often cause viral fitness losses. This study is to investigate whether strongly selected mutations that are not associated with immune responses result in fitness losses.

**Results:**

Strongly selected mutations were identified by analyzing 5′-half HIV-1 genome (*gag/pol*) sequences from longitudinal samples of subject CH0131. The K43R mutation in the *gag* gene was first detected at day 91 post screening and was fixed in the viral population at day 273 while the synonymous N323tc mutation was first detected at day 177 and fixed at day 670. No conventional or cryptic T cell responses were detected against either mutation sites by ELISpot analysis. However, when fitness costs of both mutations were measured by introducing each mutation into their cognate transmitted/founder (T/F) viral genome, the K43R mutation caused a significant fitness loss while the N323tc mutation had little impact on viral fitness.

**Conclusions:**

The rapid fixation, the lack of detectable immune responses and the significant fitness cost of the K43R mutation suggests that it was strongly selected by host factors other than T cell responses and neutralizing antibodies.

## Background

After infection, human immunodeficiency virus type 1 (HIV-1) rapidly evolves into a quasispecies population due to the high level of mutability of the virus and host selection pressure. Some of the most strongly selected mutations are associated with CD8^+^ T cell responses [1–5] or neutralizing antibodies (nAbs) [6, 7]. By precisely determining T cell responses using peptides based on the autologous transmitted/founder (T/F) virus proteome sequences in each infected individual and analyzing longitudinal near full-length HIV-1 genome sequences obtained by single genome amplification (SGA), we previously found that some predominant or fixed mutations were associated with T cell responses [5, 8]. However, we also observed that about one-third of rapidly predominating mutations were not associated with cytotoxic T lymphocyte (CTL), especially for those mutations not in the *env* gene [8]. This indicates that host factors other than immune responses contribute to the selection of those mutations. Investigating the landscape of host selection pressure exerting on the HIV-1 genome will provide a better understanding of the complex virus-host interactions.

Mutations that have a significant fitness cost predominate because they have a replicative advantage over the wild type virus under strong host selection pressure. Mutations selected by CTL responses generally impair viral fitness [9–13]. Fitness costs of immune escape mutations can have important implications for viral pathogenesis, transmission and vaccine development [14–21]. By introducing select mutations into their cognate T/F viral genomes, fitness change can be precisely determined by a competitive fitness assay. Our previous studies showed that both mutations selected by T cell and nAb responses significantly affected the fitness of the T/F viruses [22–25]. However, whether mutations that are not associated with immune selection can affect viral fitness has not been studied.

Our previous analysis of longitudinal 5′-half genome sequences obtained by SGA from subject CH0131 showed that strong selection pressure from the host drove some mutations to be rapidly fixed in the viral population within 2 years of infection [8]. We showed that mutations at two sites were selected by T cell immune responses [8] while one was a reversion mutation in the TW10 epitope [24]. In this study, we found that the earliest mutation K43R in the *gag* gene in CH0131 was strongly selected and rapidly predominated in the viral population within 6 months of infection. No conventional or cryptic T cell responses were detected targeting this mutation site. However, the mutation caused a significant fitness loss. Thus, our results demonstrated that host factors other than adaptive immune responses can also have strong selection pressure on viral evolution and drive genetic diversification of HIV-1 in vivo.

## Results

### Strongly selected mutations are identified in the 5′-half genome

**Fig. 1:**
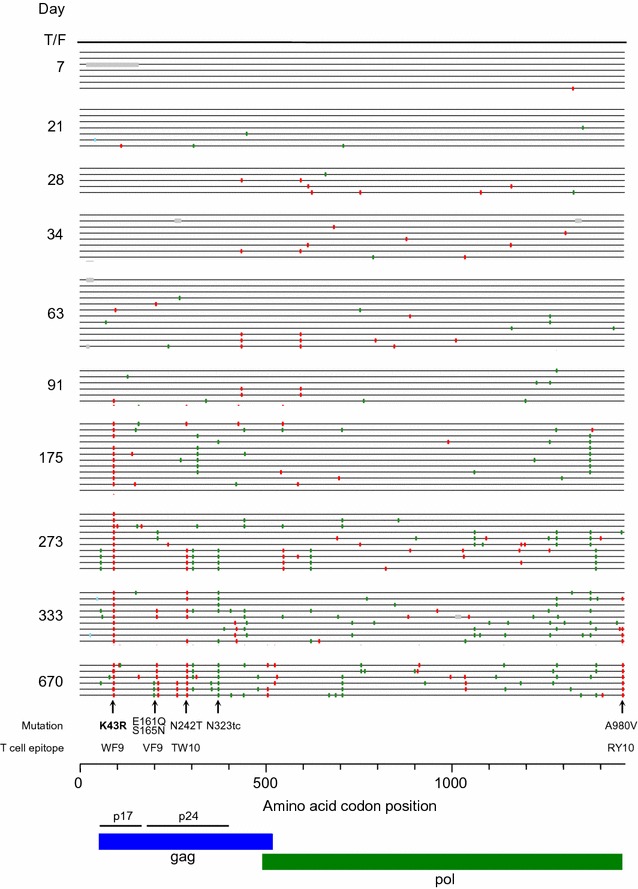
Highlighter plot of longitudinal 5′-half genome sequences. The single genome amplification (SGA) method was used to infer the transmitted/founder (T/F) 5′-half genome sequence from the screening sample and determine mutations in longitudinal samples. A highlighter plot denotes the location of synonymous (green tick), non-synonymous (red ticks), and deletion (grey ticks) mutations in each SGA-derived sequence from different time points compared to the T/F virus sequence. The positions of predominant and fixed mutations as well as related T cell epitopes are indicated on the bottom. Days post screening are indicated on the left

Analysis of longitudinal 5′-half genome (*gag/pol)* sequences from subject CH0131 (HLA A*29:01, A*23:01, B*45:01, B*15:03, Cw*02:02, Cw*06:02) identified mutations that were fixed at five sites in the viral population by day 670 post screening (Fig. 1). Mutations (K43R, E161Q/S165N, N242T and N323tc) were at four sites in the *gag* gene and one mutation (A980V) was at the end of the *pol* gene. Using autologous overlapping peptides based on the T/F whole proteome sequence, we previously detected CD8^+^ T cell responses targeting two T cell epitopes VF9_156–164_ and RY10_978–986_ [8]. By day 670, mutations E161Q/S165N and A980V in VF9_156–164_ and RY10_978–986_ epitopes were fixed in the viral population, suggesting that these mutations were selected by the T cell responses (Fig. 1). However, no T cell responses were detected for the regions containing the other three fixed mutations. The N242T mutation in the TW10_240–249_ epitope is a reversion mutation [26, 27] since the subject CH0131 did not have the HLA B*57 allele and was infected with the escape mutant virus (Fig. 1) [24]. The N242T mutation alone had no detectable impact on fitness of the cognate T/F virus [24].

**Fig. 2:**
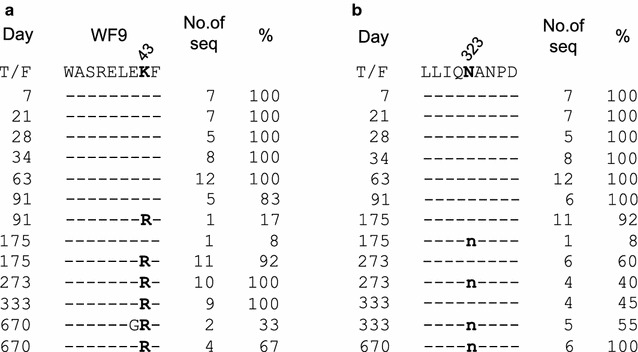
Fixed mutations in the *g* *ag* gene. The fixed K43R (**a**) and N323tc (**b**) mutations are indicated with bold letters among longitudinal sequences from subject CH0131. Dashes indicate amino acids identical to the T/F virus sequence

The K43R mutation, located in an HLA-B*35-restricted WF9_36–44_ epitope (WASRELERF) in Gag [28], was the first detected mutation (day 91). It became predominant (83%) at day 175 and was fixed in the viral population at days 273–670. This indicated that this mutation was strongly selected in the host (Figs. 1 and 2a). The synonymous N323tc mutation in Gag was first detected at day 175 and fixed at day 670 (Figs. 1 and 2b).

### The K43R and N323tc mutations are not selected by T cell responses

**Fig. 3:**
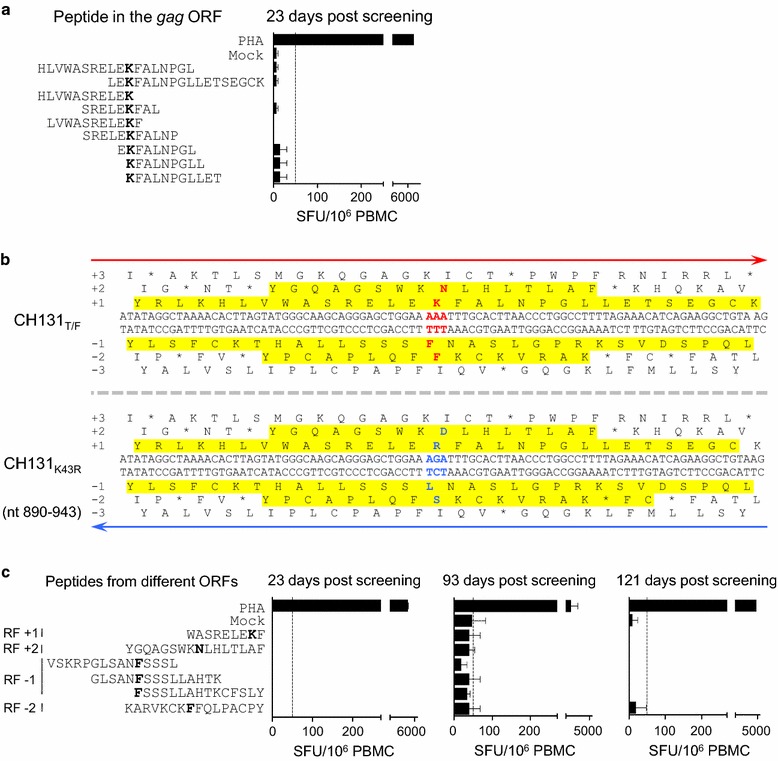
Detection of T cell responses targeting the region containing the K43R mutation. **a** Peptides containing the K43R mutation in the *gag* open reading frames (ORF) were used to detect T cell responses by the IFN-γ ELISpot assay. The dotted line indicates the threshold of a positive ELISpot response. **b** Identification of potential cryptic epitopes in all ORFs at the K43R mutation site. All potential ORFs on both DNA strands were compared between the T/F and mutant sequences at the mutation sites. Only a potential ORF at least 11 amino acids long is considered. Compared to the T/F sequences, the ORFs with amino acid substitutions (shown in red and blue in T/F sequences and the mutant sequences, respectively, at the mutation sites) are indicated in yellow. **c** T cell responses were detected with PBMCs from various days post screening and peptides containing potential cryptic epitopes at the K43R site by the IFN-γ ELISpot assay

Since no T cell responses were detected against the WF9 epitope using 18-mer peptides with 10 amino acid overlap in our previous study [8], we further determined T cell responses using various shorter 9-mer peptides containing the K43 site by ELISpot. None of the peptides were recognized by the T cells (Fig. 3a). To determine if it was selected by cryptic T cell epitopes [29, 30] from alternative expression products in the HIV-1 genome, we examined all potential open reading frames (ORFs) and found that the A → G mutation in the K43 codon (AAA) resulted in amino acid substitutions in three alternative ORFs (Fig. 3b). To determine if these potentially coded proteins were targeted by T cell responses, we tested all possible peptides containing the K43R mutation for their ability to stimulate T cell responses. None of these peptides could be recognized by T cells from days 23, 93 and 121 (Fig. 3c). These results demonstrated that the K43R mutation was not selected by classic or cryptic T cell responses.

**Fig. 4:**
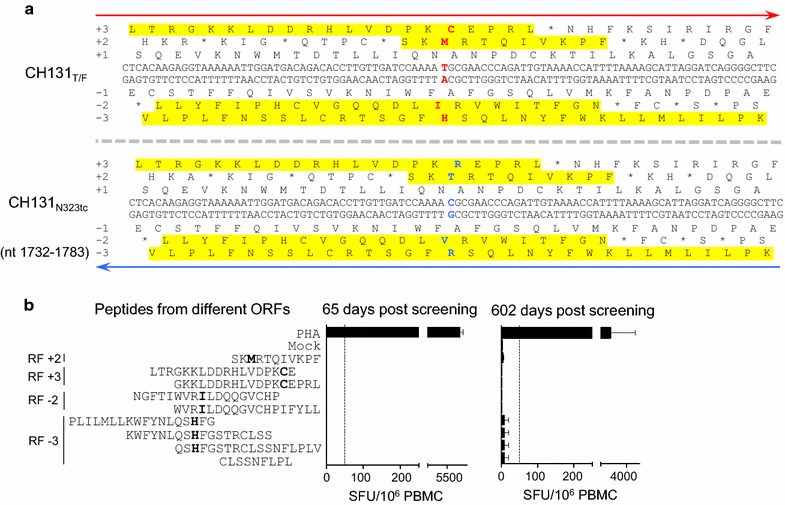
Detection of T cell responses targeting the region containing the N323tc mutation. **a** Identification of potential cryptic epitopes in all ORFs at the N323tc mutation site. All potential ORFs on both DNA strands were compared between the T/F and mutant sequences at the mutation sites. Only a potential ORF at least 11 amino acids long is considered. Compared to the T/F sequences, the ORFs with amino acid substitutions (shown in red and blue in T/F sequences and the mutant sequences, respectively, at the mutation sites) are indicated in yellow. **b** T cell responses were detected with PBMCs from various days post screening and peptides containing potential cryptic epitopes at the N323tc site by the IFN-γ ELISpot assay. The dotted line indicates the threshold of a positive ELISpot response

T cell responses targeting the site containing the synonymous N323tc mutation were also not detected with autologous Gag peptides [8]. To further investigate if this mutation was selected by cryptic T cell responses, we tested nine peptides from four alternative ORFs (Fig. 4a) to determine if these potentially coded proteins were targeted by T cell responses. ELISpot analysis showed that none of those peptides were recognized by the T cells from days 65 and 602 (Fig. 4b). This suggests that the N323tc mutation was also not selected by classic or cryptic T cell responses.

### The K43R mutation causes significant fitness loss

**Fig. 5:**
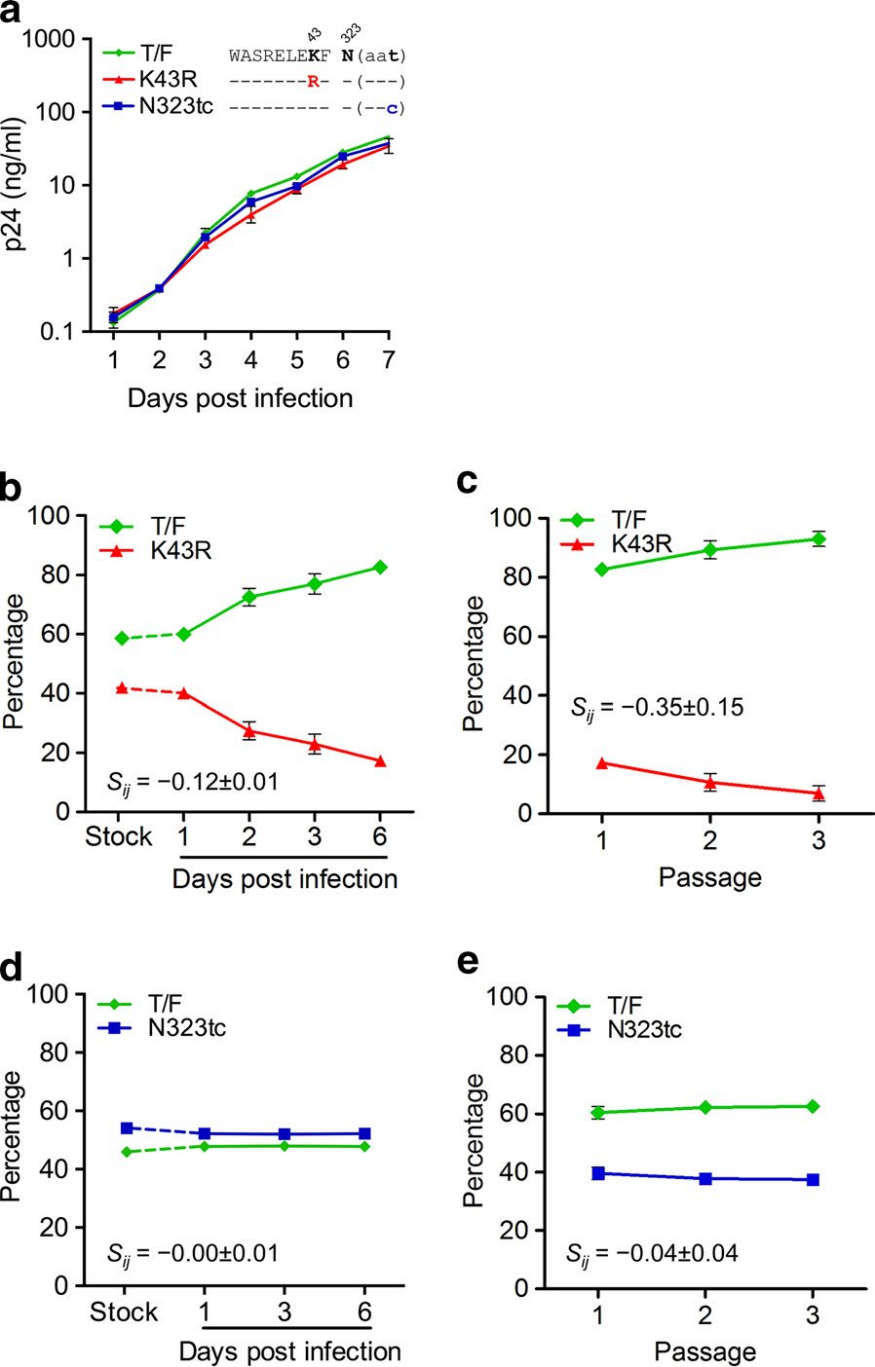
Fitness cost of the K43R mutation. **a** Replication of CH0131 T/F virus, the K43R mutant and the N323tc mutant were determined by culturing each virus independently in primary CD4^+^ T cell. Relative fitness costs of the K43R mutant (**b**) and the N323tc mutant (**d**) were determined by measuring the proportions of both compared viruses in the same cell culture using the competitive PASS fitness assay. Fitness costs of the K43R mutant (**c**) and the N323tc mutant (**e**) were determined after three passages. All experiments were carried out in triplicate. Mean ± standard deviation is shown

One important parameter to evaluate whether a mutation is selected by a host is to determine if it causes fitness loss. A fixed mutation with significant fitness loss will indicate a strong host selective pressure. To determine if the K43R and N323tc mutations affected viral fitness, we introduced each mutation into their cognate T/F infectious molecular clone (IMC) [24] and determined their fitness costs. The T/F and the two mutant viruses replicated equally well in primary CD4^+^ T cells when cultured individually (Fig. 5a). When the K43R mutant was cultured together with the T/F virus, the proportion of the K43R mutant gradually decreased while the T/F virus gradually increased in the culture. Our model analysis showed that the K43R mutant was 12% less fit than the cognate T/F virus (− 12% ± 1%; *p* = 0.003 by *t* test) (Fig. 5b). Additional passages showed that the proportion of the K43R mutant continuously decreased after each passage and was almost completely replaced by the T/F virus by passage 3 (Fig. 5c). However, no fitness differences between the N323tc mutant and the T/F virus were observed in the single passage assay (− 0.00% ± 0.01%) (Fig. 5d). After three passages, the proportion of the N323tc mutant only slightly decreased (Fig. 5e). These results demonstrated that the K43R mutation caused a significantly fitness loss while the N323tc mutation had little impact on viral fitness.

## Conclusions

The K43R mutation quickly emerged and was rapidly fixed in the viral population after infection in CH0131, suggesting that it was strongly selected in the host. Because no conventional or cryptic T cell responses were detected for the regions containing the K43R mutation and that the Gag protein does not elicit neutralizing antibodies, the strong selection pressure on the K43R mutation could not be from adaptive T and B cell responses. We have shown clearly that strong immunodominant T cell responses exerted the strongest and most rapid selection pressure in our previous study [8]. Using the same approach, we showed that T cell responses to the WF9 epitope were absent or at least below the level of detection in this highly sensitive assay. Therefore, if there were T cell responses targeting the K43R site that were undetectable (and far from immunodominant), it would be difficult to envision how it could exert any significant pressure to select such a rapid escape, with a fitness loss.

T cell responses were also not found to target the N323tc mutation site, but the mutation had little impact on viral fitness. The majority of the N323tc mutation was found in the sequences that had the K43R and N242T mutations (Fig. 1). Thus, the N323tc mutation was not likely selected by the host, but was instead a concurrent mutation together with the N242T or K43R mutation.

Analysis of the *gag* gene sequences in the Los Alamos HIV sequence database (https://www.hiv.lanl.gov) showed that R43 was present in 97.3% of the distinct viruses while K43 only accounted for 2.2% of the population. Since the K43R mutation was found in the WF9 epitope restricted by the B*35 allele that CH0131 did not carry, it was mostly likely a reversion mutation (to the consensus of subtype C viruses). However, the significant fitness loss of the K43R reversion mutation indicates that it was not simply reverted back to the consensus state due to the lack of allele-specific selection pressure, but instead it was strongly selected in vivo. We have previously found that three other reversion mutations (V247I and T242N in Gag as well as I64T in Tat) also did not cause detectable fitness differences [24, 25]. These results demonstrate that reversion mutations alone do not necessarily lead to more fit viruses. The other possibility is that K43R is a compensatory mutation. It is known that the fitness loss caused by some mutations can be restored by compensatory mutations [10, 12, 16, 22, 31, 32]. This is also not likely since the K43R mutation was the very first mutation that selected and fixed in the viral population before other mutations were detected in the 5′-half genome. Mutations in *vpu*, *env* and *nef* were also detected in the 3′-half genome sequences when the K43R mutation was detected [8]. However, it is unlikely that a mutation in the *gag* gene can compensate fitness losses due to mutations in those genes. K43R should not be a compensatory mutation for any mutations in LTR because such mutations were not detected in the viral genome. Most importantly, since the role of compensatory mutations is to restore fitness loss caused by other mutations, they alone should not result in fitness losses. Otherwise, they will cause additional fitness damage.

Genetic polymorphisms at many sites in the viral genome have been found to be associated with NK cell responses [33, 34] and anti-Gag antibody responses [35, 36]. However, specific mutations associated with selection pressure from NK cells or anti-Gag Ab responses have not been clearly identified as those mutations selected by T cell responses for in-frame or cryptic epitopes or Ab responses against epitopes in Env [1–7, 37]. Thus, it is unlikely that the K43R mutation was selected by NK cell responses and anti-Gag antibody responses. It will be important to determine if the K43R mutation or similar mutations are selected by NK cell responses and anti-Gag antibody responses or by T cell responses targeting to the spliced epitopes [38]. While only the K43R mutation was fully documented here, we found other possible examples of selected escapes that were not within an epitope stimulating a measurable T cell response in our previous studies [5, 8]. Therefore, this could be a more widespread phenomenon and this study could raise this as an issue for further study. Similar mutations should be studied further to increase our understanding on how such mutations are strongly selected by the host and if the significant fitness loss caused by the mutation is associated with lower viral loads due to the significantly reduced viral replicative capacity.

## Methods

### Site-directed mutagenesis

The K43R and N323tc mutants were obtained by introducing each mutation into the cognate T/F IMC as previously described [24].

### Ex vivo IFN-γ ELISpot assay

T cell responses to the targeting sites at various days post screening in CH0131 were determined using an in vitro IFN-γ ELISpot assay as we previously described [5, 8]. Peripheral blood mononuclear cells (PBMCs) were obtained through leukophereses from healthy donors under clinical protocols approved by the Duke University Institutional Review Board. ELISpot data are expressed as the mean spot forming units (SFU) per million PBMCs (SFU/10^6^ PBMC) ± SEM. Positive T cell responses were defined as: ≥ 50 SFU/million PBMC and > 4 times above background. All assays were performed in triplicate with a peptide concentration of 2 μg/ml.

### PASS fitness assay

PBMCs were obtained through leukophereses from healthy donors under clinical protocols approved by the Duke University Institutional Review Board. CD4^+^ T cells were purified with the QuadroMACS Separator (Miltenyi Biotec, Auburn, CA) and the CD4^+^ T cell Isolation Kit II (Miltenyi Biotec, Auburn, CA). CD4^+^ T cells (5 × 10^5^ cells) were infected with the mixture of two compared viruses (2.5 ng of p24 for each virus) both in the single passage and through multiple passages as described before [22]. The viral cDNA was synthesized with the primer low3 (5′-TTTTTCCTAGGGGCCCTGCAATTT-3′; nucleotide (nt) 1998–2021 in HXB2). The PASS amplifying primers were the forward primer M6F2 (5′-Acry-CTCGACGCAGGACTCGGCTTGCTG-3′; nt 685–708) and the reverse primer M6R2 (5′-TCCTCCCACTCCCTGACATGCTGTCATCATTTC-3′; nt 1822–1854). The bases were determined with sequencing primers juxtaposed to the mutation sites. All assays were performed in triplicate.

### Modeling relative fitness and statistical analyses

The relative fitness is determined by measuring the replication slope of each virus in the culture over time using the mathematical model and the statistical analysis was performed as previously described [22, 24].

## Abbreviations

HIV-1: human immunodeficiency virus type 1
nAb: neutralizing antibody
T/F: transmitted/founder
SGA: single genome amplification
CTL: cytotoxic T lymphocyte
HLA: human leukocyte antigen
IMC: infectious molecular clone
PBMC: peripheral blood mononuclear cell
